# Impact of Intensified Chemoimmunotherapy Regimens in Aggressive Non‐Hodgkin Lymphoma on Reproduction Patterns

**DOI:** 10.1111/ejh.70303

**Published:** 2026-08-25

**Authors:** Stefanie Antonilli, Anna Marklund, Joshua Entrop, Caroline E. Dietrich, Ingrid Glimelius, Tarec C. El‐Galaly, Alexander Fosså, Harald Holte, Karin E. Smedby, Knut B. Smeland, Sandra Eloranta

**Affiliations:** ^1^ Division of Clinical Epidemiology, Department of Medicine Solna Karolinska Institutet Stockholm Sweden; ^2^ Department of Reproductive Medicine, Division of Gynecology and Reproduction Karolinska University Hospital Stockholm Sweden; ^3^ Red Door Analytics AB Stockholm Sweden; ^4^ Department of Immunology, Genetics and Pathology, Cancer Precision Medicine Uppsala University Uppsala Sweden; ^5^ Department of Clinical Epidemiology Aarhus University Hospital Aarhus Denmark; ^6^ Department of Molecular Medicine Aarhus University Hospital Aarhus Denmark; ^7^ Department of Hematology Aarhus University Hospital Aarhus Denmark; ^8^ Department of Oncology Oslo University Hospital Oslo Norway; ^9^ Department of Hematology Karolinska University Hospital Stockholm Sweden

**Keywords:** cancer survivorship, chemotherapy, epidemiology, lymphoma, parenthood

## Abstract

Concerns about treatment‐related fertility impairment are common among young adult lymphoma survivors. Evidence on the impact of treatment for aggressive non‐Hodgkin lymphoma (NHL) on future childbearing is limited, although many individuals of reproductive age are affected. This matched cohort study identified male (18–52 years) and female (18–40 years) patients with aggressive NHL between 2007 and 2018 in Sweden and the south‐east health‐care region in Norway, and matched lymphoma‐free comparators. Adjusted hazard ratios (aHRs) comparing childbirth rates in patients treated with primary intensive chemoimmunotherapy (CIT) (e.g., R‐CHOEP and R‐DA‐EPOCH) to standard‐of‐care (R‐CHOP), and to comparators, were estimated using Cox regression. Among 1037 young survivors of aggressive NHL, childbirth rates did not differ significantly by treatment intensity (intensive vs. standard CIT) in females (aHR: 0.61, 95% CI: 0.32–1.13) or males (aHR: 1.20, 95% CI: 0.68–2.09), overall or when stratified by time since diagnosis (< 3 and 3–10 years). However, females treated with intensive CIT had lower childbirth rates than comparators during the first 3 years after diagnosis (aHR: 0.52, 95% CI: 0.30–0.88), but not thereafter (aHR: 1.06, 95% CI: 0.57–1.96). A similar pattern was not seen in males. In Sweden, eight children (three with support from assisted reproductive techniques) were born after stem cell transplantation. In conclusion, childbirth rates did not differ by CIT intensity among survivors of aggressive NHL. Although rates were temporarily reduced in females during the treatment and recovery period after diagnosis, subsequent rates were similar to comparators. These findings provide reassurance, while highlighting the importance of early and ongoing fertility counseling.

## 1 Introduction

Lymphomas constitute a mixed group of malignancies of B‐, T‐, or NK‐cell origin. Traditionally, malignant lymphomas have been classified as Hodgkin and non‐Hodgkin lymphomas (NHL), although non‐Hodgkin subtypes are heterogeneous in terms of biology, clinical manifestation, treatment protocols, and prognosis [1]. Approximately 1900 and 1250 new cases of malignant lymphomas are diagnosed each year in Sweden and Norway, respectively. Of these, close to 90% are of an NHL subtype.

First‐line treatment guidelines for NHL are relatively harmonized in the Scandinavian countries. Alkylating agents, various other chemotherapeutic agents, and gonadal radiation are known to induce infertility in a dose‐dependent manner [2, 3]. Patients diagnosed with the more common aggressive subtypes are mostly treated with the anthracycline‐based regimen CHOP (cyclophosphamide, doxorubicin, vincristine, and prednisolone), but more intensive combinations such as CHOEP (with addition of etoposide), or treatment schedules with prolonged infusion times as in dose‐adjusted (DA)‐EPOCH (etoposide, prednisone, vincristine, cyclophosphamide, and doxorubicin). For mature B‐cell lymphomas, rituximab (R) is added to these regimens. Standard anthracycline‐containing regimens such as 6 cycles of CHOP carry low (< 20%) to intermediate (40%–60% among women treated at ages > 35) risk of long‐term gonadal dysfunction in women [4]. Most male patients recover their spermatogenesis within 2 years after treatment, and the majority of premenopausal women resume ovarian function, especially if aged under 35 at the time of treatment when no more than six cycles of CHOP are given with or without etoposide [5, 6, 7, 8]. For DA‐EPCOH, limited data suggest preservation of ovarian function in most female patients treated at a younger age, but data for male survivors are scarce [9]. Burkitt lymphoma, a rare type of aggressive B‐cell NHL, is typically treated with intensified multiagent regimens with R and higher doses of antimetabolites such as methotrexate and cytarabine, for example, BFM (Berlin‐Frankfurt‐Munster) and GMALL (German Multicenter Study Group for Acute Lymphoblastic Leukemia) regimens. The degree of fertility preservation (FP) in adults treated with these regimens is not well known. In contrast, further intensification as myeloablative high‐dose chemotherapy or other intensive regimens followed by autologous or allogeneic hematopoietic stem cell transplant (SCT) used as consolidation after successful first‐line treatment of some NHL subtypes or as salvage therapy markedly increases the long‐term infertility risk [10]. While the use of radiotherapy in the treatment of NHLs is becoming increasingly rare in the era of positron emission/computerized tomography, it is occasionally still used as part of combined modality treatment in early stages or as consolidation treatment against lesions with residual fluorodeoxyglucose uptake, thereby adding to the cumulative risk of infertility when the field of radiation includes gonads [6, 11, 12].

Concerns about the impact of cancer treatment on subsequent fertility are common among young adult lymphoma survivors, and correct information is important for survivors' future family planning [13, 14]. Compared to data on survivors after Hodgkin lymphoma, evidence on the impact of cancer treatment on childbearing after NHL is scarce, possibly due to a higher average age at diagnosis in NHL than in Hodgkin lymphoma. However, the absolute number of individuals in reproductive age affected by NHL is still substantial due to the overall higher incidence of NHL compared to Hodgkin lymphoma. This highlights the need for further investigations on the effect of NHL treatment on future childbearing in NHL survivors.

In this study, we therefore investigate how childbirth rates among males and females previously diagnosed with aggressive NHL are associated with the different chemoimmunotherapy (CIT) regimens used as part of primary treatment in a population‐based cohort from Sweden and Norway. Of particular interest was to study reproduction patterns after treatment regimens of different intensities as used in first line.

## 2 Materials and Methods

### 2.1 Study Population

This is a population‐based matched cohort study for which we identified men aged between 18 and 52 years, and women between 18 and 40 years diagnosed with aggressive NHL including the following subtypes: Diffuse large B‐cell lymphoma, mantle cell lymphoma, Burkitt lymphoma, primary mediastinal B‐cell lymphoma, aggressive B‐cell lymphomas not otherwise specified, and aggressive T‐cell lymphomas. A detailed description of the histopathological codes used to identify these subtypes is listed in Table S1.

For the Swedish cohort, data for all patients diagnosed between January 2007 through June 2018 were obtained via *LymphomaBaSe*, a research database originating from the Swedish Lymphoma Register [15] and matched to several national health data registers including the patient register (for information on comorbidities and medical history), medical birth register (for childbirths), the cause of death register (for mortality data), the National Quality Registry for Assisted Reproduction (Q‐IVF) (for fertility treatments), the total population register (for migration data) and the longitudinal integrated database for health insurance and labor market studies (for education data). For the Norwegian cohort, we extracted data from a clinical lymphoma database that includes information on all adult patients diagnosed at Oslo University Hospital, the main referral hospital for lymphoma patients in the health‐care region south‐east in Norway, with a catchment area encompassing approximately 55% of the Norwegian population. Patients with a diagnosis between January 2007 and June 2016 were included and linked to the Norwegian medical birth register and cause of death register. No data on assisted reproduction was available in Norway.

Patients with a recorded history of solid organ transplantation or allogeneic SCT (*n* = 17) or HIV/AIDS (*n* = 36) prior to diagnosis of NHL were excluded. Patients with non‐standard treatment protocols for aggressive NHL were also excluded (*n* = 11) (Table S2). In both countries, each lymphoma patient was then matched on sex, birth year, and time of diagnosis to 10 (Sweden) and 5 (Norway) lymphoma‐free comparators from the general population, respectively.

Follow‐up for childbirth started 9 months after primary treatment completion, with the assumption that first‐line treatment for aggressive lymphoma, including posttreatment response assessments, lasts approximately 9 months. Thus, included patients had to be alive at 18 months of their initial diagnosis (or matching date for comparators). For this reason, 116 patients who died prior to the start of follow‐up were excluded along with their matched comparators. There were no patients who had a child within this 18‐month window. In total, our pooled cohort of Swedish and Norwegian patients included 1172 patients and 10 340 comparators (Figure S1).

### 2.2 Exposure Definition

The main exposure was defined by primary treatment. Standard CIT comprised up to six cycles of (R)‐CHOP, without intercalated or subsequent systemic central nervous system (CNS) prophylaxis. Intensive CIT included regimens typically used for high‐risk lymphomas, such as (R)‐CHOP with systemic CNS prophylaxis, (R)‐CHOEP, (R)‐DA‐EPOCH, (R)‐Hyper‐CVAD (cyclophosphamide, vincristine, doxorubicin, and dexamethasone), other high‐dose methotrexate–based regimens, NLG‐MCL2/3 protocols for mantle cell lymphoma, and BFM/GMALL‐type protocols for Burkitt lymphoma (see Table S2 for a complete list). Consolidative SCT, that is, autologous SCT performed within 1 year of diagnosis of mantle cell lymphoma, T‐cell lymphoma, or transformed low‐grade B‐cell lymphomas, was not included as a separate category in the main exposure definition, but was presented for descriptive purposes in relation to childbirths with or without assisted reproduction among Swedish patients. We did not have information on relapse or relapse treatments other than SCT. Allogeneic SCT and all remaining (i.e., not consolidative) autologous SCTs performed during follow‐up were classified as relapse treatment in the descriptive subgroup analysis. Chimeric antigen receptor T‐cell therapy was used only to a very limited extent during the study period and exclusively as third‐line treatment.

### 2.3 Statistical Methods

Patients and comparators were followed from the index date (18 months after diagnosis or matching date for comparators) until the date of first childbirth, date of death, administrative censoring (end of 2019 in Sweden and 2017 in Norway) or a maximum of 10 years, whichever came first. The time scale was time since diagnosis with delayed entry at 18 months. Comparators were additionally censored if diagnosed with any form of lymphoma during follow‐up.

In the primary analysis, the aim was to compare first childbirth rates among aggressive NHL survivors by lymphoma treatment intensity (standard vs. intensive CIT). Individuals with missing information on treatment (*n* = 119) or stage (*n* = 16) were included in the descriptive summary of the cohort for completeness but not in regression models. Standardized mean differences, comparing patients and comparators with respect to their distribution of demographic variables at baseline, were calculated.

Cox regression was used to estimate adjusted hazard ratios (aHRs) and 95% confidence intervals (CIs) comparing the treatment groups. For confounding control, we assumed a directed acyclic graph (DAG) a priori for the causal reasoning (Figure S2). Based on the DAG, the Cox regression model was adjusted for age at diagnosis (18–25, 26–40, and 41–52 years), lymphoma subtype, and stage (I–II and III–IV) in the main analysis. The model was further stratified by country to allow for heterogeneous baseline hazard functions in Sweden and Norway. Separate models were estimated for males and females to allow for effect modification by sex.

To account for nonproportional hazards, follow‐up time was split into two time intervals (< 3 years and 3–10 years after diagnosis), yielding two separate estimates of the HRs.

As secondary analysis, we estimated the cause‐specific cumulative incidence of first childbirth non‐parametrically in the presence of the competing risk of death by sex and treatment intensity both in NHL patients and the matched comparators using the Aalen‐Johansen estimator. Gray's test was used to formally test for differences in cumulative incidence functions. Among males, this analysis was restricted to men diagnosed at ages 18–40 only to control for confounding by age.

Finally, we performed three pre‐specified supplementary analyses for descriptive purposes, where we

1. described the number of children born during follow‐up, in total and with the use of assisted reproductive techniques (ART) in Sweden, stratified by lymphoma treatment intensity, including the use of SCT;
2. repeated the main analysis in the Swedish patient population without history of autoimmune diseases, additionally adjusting for highest attained education (< 13 and ≥ 13 years) as this information was not available from Norway, with censoring also for emigration;
3. repeated the analysis excluding patients who received treatment protocols according to the European Intergroup EURO‐LB02 and NOPHO ALL trials due to the longer duration of these regimens.

The statistical analyses were performed using Stata (StataCorp. 2023. Stata 18. Statistical software. StataCorp LLC).

## 3 Results

We identified a total of 1172 patients with aggressive NHL, of whom 910 (78%) were diagnosed in Sweden and 262 (22%) in Norway (Table 1). Among patients aged 18–40 years at diagnosis, 59% (*n* = 348) were males and 41% (*n* = 243) were females. In addition, we included 581 males who were 41–52 years old at diagnosis. Among all patients, the median age was 40 years (42 for males and 32 for females). By subtype, the median age was highest for patients with mantle cell lymphoma (48 years), followed by diffuse large B‐cell lymphoma (41 years), T‐cell lymphomas (40 years), and Burkitt lymphoma (32 years).

**TABLE 1:** Characteristics of the cohort of Swedish and Norwegian lymphoma populations and their matched general population comparators stratified by childbirth (no/yes) during follow‐up.

Characteristic		Lymphoma patients, *N* (%)		Comparators, *N* (%)	
		No child during follow‐up	Childbirth during follow‐up	No child during follow‐up	Childbirth during follow‐up
Total	All patients	1040 (89)	132 (11)	9014 (87)	1326 (13)
Country	Sweden	805 (88)	105 (12)	7842 (87)	1199 (13)
	Norway	235 (90)	27 (10)	1172 (90)	127 (10)
Sex	Male	847 (91)	82 (9)	7391 (91)	761 (9)
	Female	193 (79)	50 (21)	1623 (74)	565 (26)
Age at inclusion (years)	18–25	89 (74)	31 (26)	785 (73)	296 (27)
	26–40	380 (81)	91 (19)	3231 (78)	925 (22)
	41–52^a^	571 (98)	10 (2)	4998 (98)	105 (2)
Age at inclusion (years)	Median	42	30	42	30
Number of children prior to inclusion	No children	419 (82)	89 (18)	3368 (81)	799 (19)
	1 child	157 (84)	30 (16)	1441 (83)	296 (17)
	> 1 child	464 (97)	13 (3)	4205 (95)	231 (5)
Inclusion year	2007–2010	353 (84)	69 (16)	2966 (82)	640 (18)
	2011–2014	388 (88)	53 (12)	3321 (86)	524 (14)
	2015–2018	299 (97)	10 (3)	2727 (94)	162 (6)
Highest attained education level (years)^b^	9 (low)	90 (94)	6 (6)	959 (90)	104 (10)
	10–12 (intermediate)	410 (91)	41 (9)	3728 (88)	498 (12)
	≥ 13 (high)	301 (84)	58 (16)	3038 (84)	593 (16)
	Missing	4 (100)	0 (0)	117 (97)	4 (3)
Pre‐existing autoimmune diseases^b^	Yes	13 (100)	0 (0)	58 (87)	9 (13)
Lymphoma subtype	DLBCL	809 (89)	99 (11)		
	MCL	60 (98)	1 (2)		
	Aggressive B cell, nos	3 (100)	0 (0)		
	BL	59 (72)	23 (28)		
	Aggressive T cell	109 (92)	9 (8)		
WHO performance status	0–1	896 (90)	105 (10)		
	2–4	97 (82)	21 (18)		
	Missing	47 (89)	6 (11)		
Lymphoma stage	Low stage (I–II)	479 (88)	66 (12)		
	Advanced stage (III–IV)	513 (90)	59 (10)		
	Missing	48 (87)	7 (13)		
Extra‐nodal disease	Yes	526 (89)	62 (11)		
Bulky disease	Yes	288 (85)	51 (15)		
Chemoimmunotherapy (CIT)	Standard	404 (89)	48 (11)		
	Intensive	531 (88)	70 (12)		
	Missing	105 (88)	14 (12)		

Abbreviations: BL, Burkitt lymphoma; DLBCL, diffuse large B‐cell lymphoma; MCL, mantle cell lymphoma; NOS, not otherwise specified.

^a^ Only males were included in this age group.

^b^ Only available for Swedish population.

During follow‐up, 132 (82 males and 50 females) had a child. The majority of these individuals had received treatment classified as intensive CIT (*n* = 70). Of the patients who had a child after treatment, 67% had no children at the time of diagnosis. By contrast, among the 1040 survivors who did not have any childbirth following diagnosis, 40% were childless at diagnosis. The majority of the children born had a parent diagnosed with diffuse large B‐cell lymphoma (*n* = 99) and Burkitt lymphoma (*n* = 23), whereas childbirths after mantle cell lymphoma (*n* = 1) and T‐cell lymphoma (*n* = 9) were less common. The distribution of demographic characteristics among patients and comparators at study inclusion was balanced (Table S3).

Comparing childbirth rates in patients treated with different chemotherapeutic regimens (median follow‐up duration: 4.5 years, standard deviation: 2.8 years), we found no statistically significant difference between patients treated with standard versus intensive CIT in either female (aHR: 0.61, 95% CI: 0.32–1.13) or male (aHR: 1.20, 95% CI: 0.68–2.09) survivors (Table 2). When, additionally, stratifying on the first < 3 or 3–10 years after diagnosis, we again did not find a significant difference by treatment intensity in either sex; females (HR_0–3 years_: 0.69, 95% CI: 0.30–1.56; HR_3–10 years_: 0.52, 95% CI: 0.22–1.26) or males (HR_0–3 years_: 0.85, 95% CI: 0.44–1.64; HR_3–10 years_: 2.29, 95% CI: 0.91–5.76). Results were robust when adjusting for education in the Swedish subpopulation without a history of autoimmune disease (Table S4), and when excluding patients treated with EURO‐LB02 (*n* = 2) and NOPHO (*n* = 5) protocols (data not shown). In terms of absolute probability of having a child, as measured by the cumulative incidence, we found no overall difference by treatment intensity among either female or male survivors (Figure 1).

**TABLE 2:** Number of childbirths and adjusted hazard ratios (aHRs) of childbearing comparing patients treated with intensive and standard chemoimmunotherapy (CIT) for aggressive lymphoma. Results are presented for models that assume proportional hazards (PHs) and for models where follow‐up has been stratified into < 3 years and > 3 years, respectively.

	Females					Males				
	No. of 1st childbirths	aHR (95% CI)				No. of 1st childbirths	aHR (95% CI)			
		PH model		Time‐stratified model			PH model		Time‐stratified model	
Exposure										
Standard CIT	22	1.00	Ref.	—	—	26	1.00	Ref.	—	—
Intensive CIT	25	0.61	(0.32–1.13)	—	—	44	1.20	(0.68–2.09)	—	—
*p* value*		0.116					0.533			
Time‐split exposure										
Standard CIT < 3 years	11	—	—	1.00	Ref.	19	—	—	1.00	Ref.
Intensive CIT < 3 years	14	—	—	0.69	(0.30–1.56)	25	—	—	0.85	(0.44–1.64)
Standard CIT > 3 years	11	—	—	1.00	Ref.	7	—	—	1.00	Ref.
Intensive CIT > 3 years	11	—	—	0.52	(0.22–1.26)	19	—	—	2.29	(0.91–5.76)
*p* value*				0.646					0.063	
Confounders										
Age at diagnosis										
18–25 years	16	2.18	(1.18–4.01)	2.18	(1.18–4.01)	14	0.92	(0.50–1.69)	0.91	(0.50–1.68)
26–40 years	31	1.00	Ref.	1.00	Ref.	48	1.00	Ref.	1.00	Ref.
41–52 years	—	—	—	—	—	8	0.07	(0.03–0.16)	0.07	(0.03–0.16)
*p* value*		0.017		0.017			< 0.001		< 0.001	
Stage										
I–II	27	1.00	Ref.	1.00	Ref.	36	1.00	Ref.	1.00	Ref.
III–IV	20	1.23	(0.67–2.26)	1.24	(0.67–2.28)	34	0.93	(0.58–1.51)	0.93	(0.57–1.50)
Missing	—	—	—	—	—	—	—	—	—	—
*p* value*		0.509		0.794			0.961		0.762	
Subtype										
DLBCL	42	1.00	Ref.	1.00	Ref.	50	1.00	Ref.	1.00	Ref.
MCL	0	*NA*		*NA*		1	0.68	(0.09–5.25)	0.66	(0.09–5.07)
BL	3	1.37	(0.40–4.62)	1.36	(0.40–4.61)	18	1.41	(0.77–2.58)	1.37	(0.75–2.52)
Aggressive B cell, nos	0	*NA*		*NA*		0	*NA*		*NA*	
Aggressive T cell	2	0.71	(0.17–2.98)	0.72	(0.17–3.06)	1	0.13	(0.02–0.98)	0.13	(0.02–0.98)
*p* value*		0.617		0.630			0.019		0.021	

*Note:* Country has been included as a stratification variable in all Cox models to allow for different baseline hazard functions.

Abbreviations: BL, Burkitt lymphoma; CI, confidence interval; DLBCL, diffuse large B‐cell lymphoma; MCL, mantle cell lymphoma; NOS, not otherwise specified.

* *p* value testing for overall significance of the covariate (Likelihood ratio tests).

**FIGURE 1:**
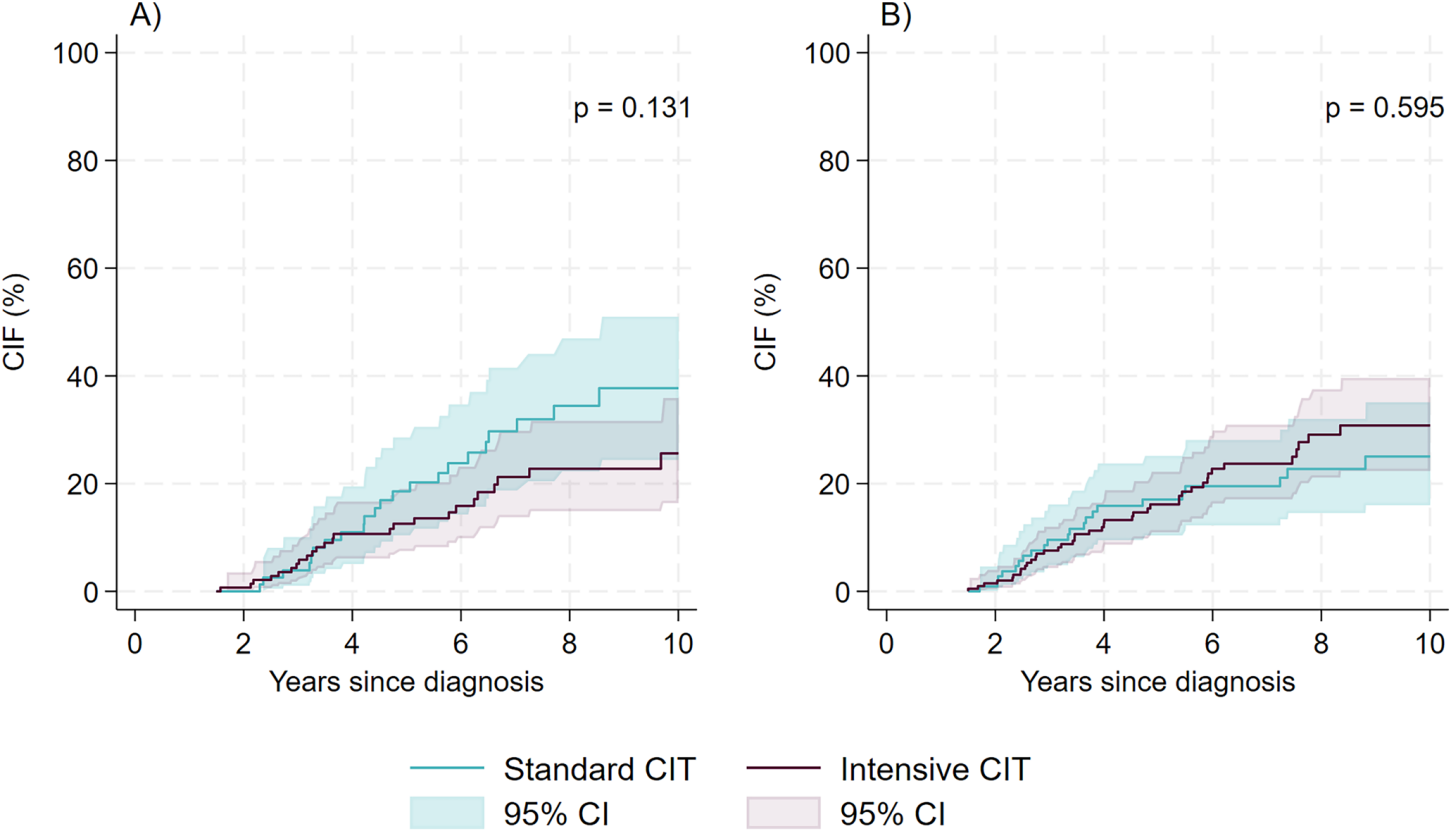
Cause‐specific cumulative incidence (CIF) and 95% confidence intervals of childbirth among female (A) and male (B) patients diagnosed with aggressive lymphoma and treated with standard and intensive chemoimmunotherapy in Sweden and Norway, stratified by sex, with *p* values for differences in CIFs. (A) Females 18–40 years. (B) Males 18–40 years.

When comparing childbirth rates by treatment intensity to matched comparators from the general population, we found childbirth rates overall to be similar among male and female lymphoma survivors and matched comparators (Table S5). However, when restricting to the first 3 years after diagnosis, females treated with intensive CIT had a lower childbirth rate than their matched comparators (HR: 0.52, 95% CI: 0.30–0.88). This also translated into an absolute reduced probability of having children when accounting for the competing risk of death (*p* value: 0.042, Figure S3). During the 3–10 years after diagnosis, the childbirth rates among female survivors treated with standard CIT and male survivors treated with intensive CIT exceeded those of the matched comparators (HR: 1.93, 95% CI: 1.04–3.55 and HR: 1.57, 95% CI: 1.00–2.50, respectively).

In Sweden, we observed a total of 105 childbirths (*n* = 44 after standard CIT, *n* = 55 after intensive CIT and 6 after treatment of unknown intensity) (Table 3). For 21 of these children (of whom 16 were born after intensive CIT), use of ART had been recorded in the Swedish Q‐IVF‐register. Of the 77 patients (N_MCL_ = 38, N_DLBCL_ = 2, N_T cell_ = 37) who underwent consolidating SCT, 2 had at least one childbirth following this treatment (one male aged 40 at diagnosis and one female aged 22, both following spontaneous pregnancies). Among 102 patients who received SCT at relapse (median age at diagnosis was 43), 1 female and 5 male survivors had at least one child post‐SCT, with ART use recorded for 3 of the men.

**TABLE 3:** Descriptive summary of patients who became parents, with or without assisted reproductive technology (ART), after completing treatment for their lymphoma. The analysis is restricted to patients in Sweden (*n* = 910).

	Number of patients, *N* (%)^a^	Number of patients with at least 1 child after treatment, *N* (%)^b^	Number of children born after ART treatment, *N* (%)^b^
Chemoimmunotherapy (CIT)			
Standard CIT	376 (41)	44 (11.7)	4 (1.1)
Intensive CIT	464 (51)	55 (11.8)	16 (3.4)
Missing CIT information	70 (8)	6 (8.6)	1 (1.4)
Stem cell transplantation (SCT)			
Consolidative treatment^c^	77 (8)	2 (2.6)	0 (−)
Relapse treatment^c^	102 (11)	6 (5.9)	3 (2.9)

Abbreviations: CIT, chemoimmunotherapy; SCT, stem cell transplantation.

^a^ Percentages are calculated based on the total cohort (*n* = 910).

^b^ Percentages are calculated from the number of patients in each treatment group. CIT categories (standard, intensive, missing) are mutually exclusive. SCT categories are not mutually exclusive (patients may also have received chemoimmunotherapy).

^c^ SCT was classified as consolidative or relapse treatment as follows: allogeneic = relapse; autologous within 1 year = consolidative for Mantle cell lymphoma/T cell and discordant Diffuse large B‐cell lymphoma; otherwise relapse. SCT include autologous (*n* = 165) and allogeneic (*n* = 12) stem cell transplantation.

## 4 Discussion

In this population‐based study combining data from Sweden and Norway, we did not find any consistent differences in childbirth rates when comparing intensive versus standard CIT in either male or female survivors of aggressive NHL. However, these results should be interpreted bearing in mind the substantial heterogeneity within the intensive CIT group, which encompasses biologically and gonadotoxically distinct chemotherapy regimens (e.g., DA‐EPOCH, Hyper‐CVAD, High‐dose methotrexate combinations, GMALL/BFM‐type regimens, and upfront autologous SCT). Notably, descriptive analyses showed that a substantial number of children were born after diagnosis and treatment of Burkitt lymphoma in adult patients. This observation likely reflects a younger patient population for this subtype (median age 32 years, 57% had no children at diagnosis) that is typically treated with intensive, but time‐limited, rituximab‐containing regimens without autologous SCT consolidation, resulting in high cure proportions and largely preserved reproductive potential over a long survivorship period. This can be contrasted to mantle‐ and T‐cell lymphomas, where a relatively low reported number of child births likely reflect the frequent use of consolidative high‐dose therapy with SCT, together with a higher median age and lower proportions of patients without children at the time of diagnosis (25% and 46% for mantle cell lymphoma and T‐cell lymphoma, respectively), all of which independently reduce the probability of a subsequent childbirth. However, we observed occasional reports of childbirths after SCT (used either as consolidative therapy or for relapse) both with or without the use of ART. Such favorable outcomes are rarely reported in the literature and may be reassuring for young patients treated for aggressive NHL.

Although childbirth rates were also broadly similar to those observed in the general population, there was some evidence that females who had undergone intensive CIT had a lower childbirth rate during the first 3 years following their lymphoma diagnosis. This corroborates our previous study on parenthood among NHL survivors in Scandinavia, where childbirth rates were similar to those of the general population, except for patients diagnosed with aggressive NHL, for whom childbirth rates were lower in the short term after diagnosis [16]. The observed reduced initial childbirth rate in females coincides with the recovery period that patients go through, with recommendations of contraceptive use after CIT and advice to delay pregnancy until completion of the period of highest relapse risk [4, 17, 18]. In the present study, a similar pattern was not evident among male survivors, who comprised nearly 80% of the study population, and the overall posttreatment parenthood rates were closer to those in the general population. An explanation for this observation for male patients may be that concerns about relapse risk or subsequent therapy are less likely to impact family decisions with an assumed healthy female partner. Other previous work in NHL where population comparators [17, 19, 20] or sibling controls [21] have been available has reported conflicting or inconclusive results in terms of overall childbirth rates and has not addressed differences by treatment.

Studies that have focused on treatment intensity in patients with aggressive NHL include a smaller German study of female NHL patients of childbearing potential treated with CHOP or CHOEP, which reported a similar and low proportion of childlessness, irrespective of first‐line treatment and sex [7]. A study of female patients treated with R‐DA‐EPOCH has further presented high menstrual recovery rates also in younger patients undergoing this regimen [9]. Yet another study investigating gonadal hormones of female survivors of NHL treated with CHOP versus CHOEP revealed low anti‐Mullerian hormone levels in female NHL patients finishing their treatment, indicating a low ovarian reserve [22]. Taken together, these previous investigations suggest that the chance of having children after finishing NHL treatment might not be permanently impacted in female NHL survivors, but rather that their fertility window is likely shortened and there may be a higher need for ART than in the general population.

Previous research on male fertility after NHL treatment is still scarce. Palotti et al. reported that males with aggressive lymphomas may exhibit poorer baseline semen quality than those with indolent subtypes [8]. Also, while standard first‐line treatments generally preserve fertility, intensified or salvage regimens are associated with a higher risk of azoospermia and impaired reproductive outcomes. Weibring et al. reported parenthood outcomes in a Swedish prospective cohort of 1378 adolescents and young adults who banked semen for FP at cancer diagnosis [23]. Among 119 men with NHL (median age 33 years), 47 later became fathers. Most conceived naturally (74.5%), while about one quarter used ART, using own or donor sperm, and 4.3% adopted.

In terms of the most intensive treatments, there were eight childbirths recorded as the first posttreatment childbirth even after SCT in the Swedish cohort. Of these children, the majority had a father previously treated for lymphoma (*n* = 6), and three were born with the help of ART. SCTs represent toxic treatments, and both the cumulative burden of gonadotoxic regimens prior to transplant, including therapy used for conditioning, is known to reduce fertility in survivors [10]. Higher age at transplant in female patients represents a risk factor for infertility after BEAM (Carmustine, etoposide, cytarabine and melphalan), the most commonly used regimen for autologous SCT in lymphoma patients in Scandinavia [24]. Moreover, other adverse outcomes after SCT, such as graft‐versus‐host disease, increased comorbidity burden, and reduced quality of life, may indirectly impact the willingness to become a parent after treatment. In our cohort, the relatively high median age at SCT (43 years) and short potential follow‐up is likely also contributing to the low number of posttreatment childbirths in this group of survivors.

In Sweden and Norway, young adult NHL patients without children have free access to FP and ART through the public health system. In some regions, however, patients with one or more children at the time of cancer diagnosis or initiation of ART are required to cover a part of or the whole cost themselves. The high access to FP and ART for post‐cancer pregnancy attempts might have compensated for any otherwise negative effects of aggressive NHL treatment on future parenthood. The importance of recognizing the potential risk of infertility associated with specific treatment modalities in patients of reproductive age, and of providing timely fertility counseling that includes available FP options is well established [4]. Updated FP guidelines are available to support clinical decision‐making [4, 18, 25, 26]. Still, studies indicate that female cancer patients receive fertility counseling and access FP less frequently than their male counterparts [27, 28]. In patients with aggressive NHL, this disparity may be particularly pronounced due to the therapeutic urgency of the disease. While sperm cryopreservation in post‐pubertal males can typically be arranged within 1–2 days of referral, oocyte or embryo cryopreservation in females generally requires a delay of 10–14 days before initiating chemotherapy, a postponement that is not always clinically feasible. Ovarian tissue cryopreservation is an alternative for women requiring urgent treatment initiation, except in rare cases where the medical condition renders laparoscopy or anesthesia unsafe. However, in aggressive NHL, rigorous analysis for specific molecular markers and molecular and histological evaluation of ovarian tissue prior to re‐transplantation is essential to minimize the risk of reintroducing malignant cells [29, 30]. Advances in oncofertility may, in the future, help overcome these practical limitations. Moreover, while FP options are not possible in all females with aggressive lymphoma prior to the start of gonadotoxic treatments, close follow‐up and monitoring may provide some survivors of aggressive lymphoma with an opportunity to have FP following treatment [31].

Our study has several strengths but also limitations. The use of detailed population‐based data from both Sweden and Norway, including matched controls, provided a sufficiently large patient population to contrast childbirth rates between regimens of different intensities used in the treatment of aggressive NHL. Still, the number of childbirths is limited, which prevented us from studying treatment protocols at a higher granularity than standard CHOP versus more intensive CIT, and we therefore cannot rule out moderate treatment‐related effects. Data on the utilization of FP options at the time of diagnosis were not available, and information on the use of ART was available only for the Swedish subset survivors and without specification regarding the use of donated gametes.

### 4.1 Conclusion and Clinical Implications

In conclusion, we found no clear differences in childbearing by CIT intensity in either male or female survivors of aggressive NHL. Although childbearing in female survivors treated with intensive CIT was significantly decreased during the first 3 years after NHL diagnosis, the probability of having children after the initial treatment and recovery period was similar to matched comparators also for this group. Moreover, our data show that children are born, spontaneously or with support from ART, also after SCT. Our findings provide some reassurance for young patients with aggressive NHL that the prospect of future parenthood may be preserved even after intensive treatments, but they also underscore the continued importance of fertility counseling and FP in this population.

## Author Contributions

Conceptualization: All. Data curation: Stefanie Antonilli, Joshua Entrop, and Sandra Eloranta, Karin E. Smedby, and Knut B. Smeland. Formal analysis: Stefanie Antonilli, Joshua Entrop, and Sandra Eloranta. Funding acquisition: Sandra Eloranta, Karin Ekström Smedby, Tarec C. El‐Galaly. Methodology: Stefanie Antonilli, Joshua Entrop, Sandra Eloranta, and Caroline E. Dietrich. Writing original draft: Sandra Eloranta, Joshua Entrop, Stefanie Antonilli, and Anna Marklund. Review and editing: All.

## Funding

The study was supported by the Swedish Cancer Society (Sandra Eloranta, 222017Pj), the Cancer Research Foundations of Radiumhemmet (Sandra Eloranta, 241142), the Nordic Cancer Union (Tarec C. El‐Galaly, A16008; Ingrid Glimelius, A16094), the Karolinska Institutet's Research Foundation (Sandra Eloranta), and FORTE (Dnr 2024‐01521, Anna Marklund).

## Ethics Statement

The study has been approved by the Regional Ethical Review Board in Stockholm (No. 2014/1017‐32) and the Regional Committee for Medical Research Ethics South East Norway (No. 2018/2209).

## Conflicts of Interest

Karin E. Smedby has received research funding from Janssen Pharmaceuticals NV and honoraria from AbbVie and Incyte. Ingrid Glimelius has received research funding from Takeda and honoraria for lectures from AstraZeneca, AbbVie, and Janssen Cilag, with all reimbursements to the department. Alexander Fosså has received research support from Takeda and Roche (outside of the submitted work), honoraria from Johnson and Johnson, Roche, Takeda, Merck Sharp & Dohme, BMS, Eusapharma, Kite Gilead, Kyowa Kirin, and SOBI, and served as a consultant for Takeda, Kite Gilead, and SOBI. Harald Holte has received research support from Novartis (outside the submitted work), served as a consultant for Serb AB and Incyte, and as a member of the Safety Committee for Genmab and Elicera Therapeutics. Anna Marklund has received honoraria for lectures from Merck and Ferring. Joshua Entrop is employed by Red Door Analytics AB, which provides consulting services to organizations in the academic, healthcare, and pharmaceutical sectors. The other authors declare no conflicts of interest.

## Supporting information


**Table S1:** Subtype definitions and categorization of patients diagnosed with aggressive non‐Hodgkin lymphoma in Sweden and Norway.
**Table S2:** Treatment exposure definitions for patients diagnosed with aggressive non‐Hodgkin lymphoma in Sweden and Norway.
**Table S3:** Distribution of demographic characteristics of patients and their matched comparators at baseline, including standardized mean differences.
**Table S4:** Number of first posttreatment childbirths and adjusted hazard ratios (aHRs) of childbearing comparing patients treated with intensive and standard chemoimmunotherapy (CIT) for aggressive lymphoma, restricted to patients diagnosed in Sweden (*n* = 814) without autoimmune diseases at baseline. Results are presented for models that assume proportional hazards (PHs) and for models where follow‐up has been stratified into < 3 years and > 3 years, respectively.
**Table S5:** Number of first posttreatment childbirths and adjusted hazard ratios (aHRs) of childbearing comparing patients treated with intensive and standard chemoimmunotherapy (CIT) for aggressive lymphoma to matched comparators.
**Figure S1:** Flow chart of the study population of patients with aggressive non‐Hodgkin lymphoma (NHL).
**Figure S2:** Assumed adjusted directed acyclic graph (DAG) of the association between treatment and childbirth in survivors of aggressive NHL, including a differentiation between childbearing behavior and fertility.
**Figure S3:** Cause‐specific cumulative incidence (CIF) of childbirth among female (A, C) and male (B, D) patients diagnosed with aggressive lymphoma and their matched comparators in Sweden and Norway, stratified by sex and treatment group with *p* values for differences in CIFs.

## Data Availability

Individual‐level data used for the analysis cannot be shared with others in accordance with the EU's GDPR. A detailed analysis plan describing the data used for this study has been pre‐registered on the Open Science Framework prior to the start of data analysis (https://doi.org/10.17605/OSF.IO/QMEZP).
