# Supplementary material for: Impact of Intensified Chemoimmunotherapy Regimens in Aggressive Non‐Hodgkin Lymphoma on Reproduction Patterns
Source: Eur J Haematol. 2026 Aug 25;117(6):1601–11. doi: 10.1111/ejh.70303 (PMC13729921; doi:10.1111/ejh.70303)
Supplement: Supplementary file 1 [file EJH-117-1601-s001.docx]

**Supplementary material for: Impact of intensified chemoimmunotherapy regimens in aggressive non-Hodgkin lymphoma on reproduction patterns**

Stefanie Antonilli^1^, Anna Marklund^2^, Joshua Entrop^1,3^, Caroline E. Dietrich^1^, Ingrid Glimelius^4^, Tarec C. El-Galaly^1,5,6,7^, Alexander Fosså^8^, Harald Holte^8^, Karin E. Smedby^1,9^, Knut B. Smeland^8^, Sandra Eloranta^1^

^1^Division of Clinical Epidemiology, Department of Medicine Solna, Karolinska Institutet, Stockholm, Sweden

^2^Department of Reproductive Medicine, Division of Gynecology and Reproduction, Karolinska University Hospital, Stockholm, Sweden.

^3^ Red Door Analytics AB, Stockholm, Sweden.

^4^Department of Immunology, Genetics and Pathology, Cancer Precision Medicine, Uppsala University, Uppsala, Sweden

^5^Department of Clinical Epidemiology, Aarhus University Hospital, Denmark

^6^Department of Molecular Medicine, Aarhus University Hospital, Denmark

^7^Department of Hematology, Aarhus University Hospital, Denmark

^8^Department of Oncology, Oslo University Hospital, Oslo, Norway

^9^Department of Hematology, Karolinska University Hospital, Stockholm, Sweden

**Supplementary material**

**SFigure1:** Flow chart of the study population of patient with aggressive non-Hodgkin lymphoma (NHL).

**SFigure2**: Assumed adjusted Directed Acyclic Graph (DAG) of the association between treatment and childbirth in survivors of aggressive NHL including a differentiation between childbearing behaviour and fertility.

**SFigure 3:** Cause-specific cumulative incidence (CIF) of childbirths among female (A, C) and male (B, D) patients diagnosed with aggressive lymphoma and their matched comparators in Sweden and Norway, stratified by sex and treatment group with p-values for differences in CIFs.

**STable1:** Subtype definitions and categorisation of patients diagnosed with aggressive non-Hodgkin lymphoma in Sweden and Norway.

| **Lymphoma subtype** |  |  |  | **ICD-O/3** | **ICD-10** | **Subtype categorisation** |
| --- | --- | --- | --- | --- | --- | --- |
| Blastic/aggressive NHL, B-cell lymphoma |  |  |  | 959133 | C85.9 | Aggressive B-cell lymphoma nos (not otherwise specified) |
| Primary mediastinal large B-cell lymphoma |  |  |  | 96793 | C83.3, .8, .9 | DLBCL |
| High-grade malignant B-cell lymphoma (NOS) |  |  |  | 96803 | C83.3, .8, .9 | DLBCL |
| T-cell /histiocyte rich B-cell lymphoma |  |  |  | 96883 | C83.3, .8, .9 | DLBCL |
| ALK-positive large B-cell lymphoma |  |  |  | 97373 | C83 (excl. C83.1) | DLBCL |
| Mantle cell lymphoma |  |  |  | 96733 | C83.1 | MCL |
| Burkitt lymphoma |  |  |  | 96873 | C83.7 | BL |
| Blastic/aggressive NHL, T-cell lymphoma |  |  |  | 959135 |  | Aggressive T-cell lymphoma |
| Sézary syndrome |  |  |  | 97013 | C84.1 | Aggressive T-cell lymphoma |
| Peripheral T-cell lymphoma, unspecified |  |  |  | 97023 | C84.4, C84.5 | Aggressive T-cell lymphoma |
| Angioimmunoblastic T-cell lymphoma |  |  |  | 97053 | C84.4, C84.5 | Aggressive T-cell lymphoma |
| Subcutaneous panniculitis-like T-cell lymphoma |  |  |  | 97083 | C84.4, C84.5 | Aggressive T-cell lymphoma |
| Anaplastic large cell lymphoma |  |  |  | 971435 | C84.6 | Aggressive T-cell lymphoma |
| Hepatosplenic T-cell lymphoma |  |  |  | 97163 | C84.4, C84.5 | Aggressive T-cell lymphoma |
| Monomorph epitheliotropic intestinal T-cell lymphoma |  |  |  | 97173 | C84.4, C84.5 | Aggressive T-cell lymphoma |
| Precursor T lymphoblastic lymphoma |  |  |  | 97293 | C84.5 | Aggressive T-cell lymphoma |
| Adult T-cell leukemia/lymphoma |  |  |  | 98273 | C84.5, C83.5 | Aggressive T-cell lymphoma |

**STable2:** Treatment exposure definitions for patients diagnosed with aggressive non-Hodgkin lymphoma in Sweden and Norway.

| **Treatment category** | **Number of patients** | **Treatment sub-category** | **Detailed treatment specification** |
| --- | --- | --- | --- |
| Standard treatment |  |  |  |
|  | 445 | Standard | (R)-CHOP (without systemic CNS prophylaxis) or FLYER protocol (4 R-CHOP + 2 R) |
| Intensive treatment |  |  |  |
|  | 303 | (R)-CHOEP | (R)-CHOEP with or without systemic CNS prophylaxis (MTX and/or cytarabin) |
|  | 24 | (R)-DA-EPOCH | (R)-DA-EPOCH |
|  | 37 | (R)-CHOP+CNS | (R)-CHOP with systemic CNS prophylaxis (MTX and/or cytarabin |
|  | 56 | (R)-Hyper-CVAD | (R)-Hyper-CVAD |
|  | 31 | (R)-HDMTX/cytarabin | MTX-based regimens (MTX, MPV, DeAngelis, Ferreri, NLG-CNS2, Rubinstein, IME) –(R), cytarabin, cytarabin /MTX |
|  | 49 | NLG-MCL | NLG-MCL2/3 protocols (R-cytarabin + ASCT, maxi-CHOP+cytarabin) |
|  | 92 | ALL-like | BFM, GMALL93 protocol, Hammersmith, EURO-LB02 protocol*, NOPHO protocol* |
| Other (excluded) |  |  |  |
|  | 11 | Non-standard | Bendamustine, Caelyx, VACOP-B, Vincristine, MTX po, PAI, Ixotene, Fludarabin |

*Longer duration treatment protocols. These are excluded in a separate sensitivity analysis to assess robustness of results.

**STable 3:** Distribution of demographic characteristics of patients and their matched comparators at baseline, including standardised mean differences.

|  |  | Lymphoma patients  Number (%) | Comparators  Number (%) | Standardized mean difference |
| --- | --- | --- | --- | --- |
| Total | All patients | 1,172 (100) | 10,340 (100) | - |
| Sex | Male | 929 (79) | 8,152 (79) | 0.0105 |
|  | Female | 243 (21) | 2,188 (21) |  |
| Age at inclusion (years) | 18-25 | 120 (10) | 1,081 (10) | 0.0074 |
|  | 26-40 | 471 (40) | 4,156 (40) |  |
|  | 41-52* | 581 (50) | 5,103 (50) |  |
| Inclusion year | 2007-2010 | 422 (36) | 3,606 (35) | 0.0364 |
|  | 2011-2014 | 441 (38) | 3,845 (37) |  |
|  | 2015-2018 | 309 (26) | 2,889 (28) |  |
| Number of children prior to inclusion | No children | 508 (43) | 4,167 (40) | 0.0618 |
|  | 1 child | 187 (16) | 1,737 (17) |  |
|  | >1 child | 477 (41) | 4,436 (43) |  |
| Highest attained education level (years)† | 9 (low) | 96 (11) | 1,063 (12) | 0.1105 |
|  | 10-12 (intermediate) | 451 (50) | 4,226 (47) |  |
|  | ≥13 (high) | 359 (39) | 3,631 (40) |  |
|  | Missing | 4 (0) | 121 (1) |  |

†Only available for Swedish population.

**STable 4:** Number of first post-treatment childbirths and adjusted hazard ratios (aHR) of childbearing comparing patients treated with intensive and standard chemoimmunotherapy (CIT) for aggressive lymphoma, restricting to patients diagnosed in Sweden (n = 814) without autoimmune diseases at baseline. Results are presented for models that assume proportional hazards (PH) and for models where follow-up has been stratified into <3 years and > 3 years, respectively

|  |  | **Females** | | | |  | **Males** | | | | |
| --- | --- | --- | --- | --- | --- | --- | --- | --- | --- | --- | --- |
|  |  | **No. of 1st childbirths during follow-up** | **aHR (95% CI)** | | |  | **No. of 1st childbirths during follow-up** | **aHR (95% CI)** | | | |
|  |  |  | PH Model | | Time-stratified Model | |  | PH Model | | Time-stratified Model | |
| **Exposure** | |  |  |  |  |  |  |  |  |  |  |
|  | Standard CIT | 20 | 1.00 | Ref. | - | - | 24 | 1.00 | Ref. | - | - |
|  | Intensive CIT | 24 | 0.56 | (0.29-1.09) | - | - | 30 | 1.19 | (0.65-2.19) | - | - |
|  | *P-value*^†^ |  | *0.089* |  |  |  |  | *0.570* |  |  |  |
| **Time-Split Exposure** | |  |  |  |  |  |  |  |  |  |  |
|  | Standard CIT < 3 yrs | 9 | - | - | 1.00 | Ref. | 17 | - | - | 1.00 | Ref. |
|  | Intensive CIT < 3 yrs | 13 | - | - | 0.71 | (0.29-1.74) | 17 | - | - | 0.87 | (0.42-1.79) |
|  | Standard CIT > 3 yrs | 11 | - | - | 1.00 | Ref. | 7 | - | - | 1.00 | Ref. |
|  | Intensive CIT > 3 yrs | 11 | - | - | 0.44 | (0.18-1.08) | 13 | - | - | 2.11 | (0.80-5.55) |
|  | *P-value*^†^ |  |  |  | *0.424* |  |  |  |  | *0.123* |  |
| **Confounders** | |  |  |  |  |  |  |  |  |  |  |
| *Age at Diagnosis* | |  |  |  |  |  |  |  |  |  |  |
|  | 18-25 years | 15 | 2.13 | (1.13-3.99) | 2.13 | (1.13-3.99) | 12 | 1.20 | (0.61-2.37) | 1.19 | (0.60-2.35) |
|  | 26-40 years | 29 | 1.00 | Ref. | 1.00 | Ref. | 35 | 1.00 | Ref. | 1.00 | Ref. |
|  | 41-52 years | - | - | - | - | - | 7 | 0.08 | (0.04-0.19) | 0.08 | (0.04-0.19) |
|  | *P-value*^†^ |  | *0.082* |  | *0.025* |  |  | *<0.001* |  | *<0.001* |  |
| *Stage* | |  |  |  |  |  |  |  |  |  |  |
|  | I-II | 26 | 1.00 | Ref. | 1.00 | Ref. | 31 | 1.00 | Ref. | 1.00 | Ref. |
|  | III-IV | 18 | 1.13 | (0.59-2.14) | 1.14 | (0.60-2.17) | 23 | 0.70 | (0.40-1.22) | 0.69 | (0.39-1.21) |
|  | Missing | - | - | - | - | - | - | - | - | - | - |
|  | *P-value*^†^ |  | *0.717* |  | *0.688* |  |  | *0.204* |  | *0.195* |  |
| *Subtype* | |  |  |  |  |  |  |  |  |  |  |
|  | DLBCL | 40 | 1.00 | Ref. | 1.00 | Ref. | 43 | 1.00 | Ref. | 1.00 | Ref. |
|  | MCL | 0 | NA |  | NA |  | 1 | 0.81 | (0.10-6.30) | 0.78 | (0.10-6.09) |
|  | BL | 3 | 1.56 | (0.46-5.29) | 1.55 | (0.46-5.26) | 9 | 0.96 | (0.44-2.11) | 0.95 | (0.43-2.08) |
|  | Aggressive B-cell, nos | 0 | NA |  | NA |  | 0 | NA |  | NA |  |
|  | Aggressive T-cell | 1 | 0.40 | (0.05-3.01) | 0.41 | (0.05-3.06) | 1 | 0.14 | (0.02-1.05) | 0.14 | (0.02-1.05)) |
|  | *P-value*^†^ |  | *0.398* |  | *0.407* |  |  | *0.062* |  | *0.065* |  |
| *Education* | |  |  |  |  |  |  |  |  |  |  |
|  | < 13 years | 15 | 1.00 | Ref. | 1.00 | Ref. | 29 | 1.00 | Ref. | 1.00 | Ref. |
|  | ≥ 13 years | 29 | 2.27 | (1.19-4.32) | 2.29 | (1.20-4.36) | 25 | 1.55 | (0.91-2.65) | 1.56 | (0.91-2.67) |
|  | *P-value* |  | *0.010* |  | *0.010* |  |  | *0.115* |  | *0.110* |  |
| Abbreviations: CI: confidence interval, DLBCL: Diffuse large B-cell lymphoma, MCL: Mantle cell lymphoma, BL: Burkitt lymphoma, nos: not otherwise specified.  †: P-value testing for overall significance of the covariate (Likelihood ratio tests) | | | | | | | | | | | |

**STable 5** Number of first post-treatment childbirths and adjusted hazard ratios (aHR) of childbearing

comparing patients treated with intensive and standard chemoimmunotherapy (CIT) for aggressive lymphoma to matched comparators.

|  | **Females** | |  | **Males** | | | |
| --- | --- | --- | --- | --- | --- | --- | --- |
|  | **No. of 1st childbirths** | **aHR (95% CI)** | | **No. of 1st childbirths** | **aHR (95% CI)** | | |
|  |  |  | |  |  | | |
| **Entire follow-up** |  |  |  |  |  |  | |
| Comparators | 533 | 1.00 | Ref. | 705 | 1.00 | Ref. | |
| All cases* | 47 | 0.80 | (0.59-1.08) | 71 | 0.91 | 0.71-1.16 | |
| *P-value**^†^ |  | *0.146* |  |  | *0.446* |  | |
| Comparators | 533 | 1.00 | Ref. | 705 | 1.00 | Ref. | |
| Standard CIT | 22 | 1.03 | (0.67-1.58) | 26 | 0.81 | 0.55-1.20 | |
| Intensive CIT | 25 | 0.67 | (0.45-1.00) | 45 | 0.98 | 0.72-1.32 | |
| *P-value*^†^ |  | *0.144* |  |  | *0.576* |  | |
| **Exposure 0-3 years** |  |  |  |  |  |  | |
| Comparators | 379 | 1.00 | Ref. | 494 | 1.00 | Ref. | |
| All cases** | 25 | 0.59 | (0.39-0.88) | 44 | 0.80 | (0.59-1.09) | |
| *P-value***^†^ |  | *0.010* |  |  | *0.165* |  |  |
| Comparators | 379 | 1.00 | Ref. | 494 | 1.00 | Ref. | |
| Standard CIT† | 11 | 0.70 | (0.39-1.28) | 19 | 0.89 | (0.56-1.40) | |
| Intensive CIT† | 14 | 0.52 | (0.30-0.88) | 25 | 0.75 | (0.50-1.12) | |
| *P-value*^†^ |  | *0.031* |  |  | *0.338* |  | |
| **Exposure 3-10 years** |  |  |  |  |  |  | |
| Comparators | 154 | 1.00 | Ref. | 211 | 1.00 | Ref. | |
| All cases** | 22 | 1.37 | (0.88-2.14) | 27 | 1.16 | (0.77-1.73) | |
| *P-value***^†^ |  | *0.168* |  |  | *0.477* |  | |
| Comparators | 154 | 1.00 | Ref. | 211 | 1.00 | Ref. | |
| Standard CIT^‡^ | 11 | 1.93 | (1.04-3.55) | 7 | 0.66 | (0.31-1.40) | |
| Intensive CIT^‡^ | 11 | 1.06 | (0.57-1.96) | 20 | 1.57 | (1.00-2.50) | |
| *P-value*^†^ |  | *0.111* |  |  | *0.079* |  | |
| * HR from a separate model including case status and age at diagnosis, stratified by country,  ** HR from a separate model including case status and age at diagnosis, stratified by country, with time-varying effect for case status (split at 3 years).  † P-value testing for overall significance of the covariate (Wald tests) | | | | | | | |
| ‡ HR from the main model including treatment category (standard, intensive, comparator) and age at diagnosis, stratified by country, with time-varying effect for treatment (split at 3 years). | | | | | | | |

**SFigure1:** Flow chart of the study population of patient with aggressive non-Hodgkin lymphoma (NHL).

SCT: stem cell transplantation.

^†^Comparators were censored at the date of any lymphoma diagnosis and subsequently included as cases if the diagnosed lymphoma met the study’s inclusion and exclusion criteria for aggressive lymphomas.

**SFigure2**: Assumed adjusted Directed Acyclic Graph (DAG) of the association between treatment and childbirth in survivors of aggressive NHL including a differentiation between childbearing behaviour and fertility.

The green node is the exposure variable, blue with a black line is the outcome variable, the other blue are the ancestors of outcome and the red node is an ancestor of exposure and outcome. White nodes are the adjusted variables, grey nodes are the unmeasured latent variables. The black edges are the biasing paths and green edges are the causal paths.

**SFigure 3:** Cause-specific cumulative incidence (CIF) of childbirths among female (A, C) and male (B, D) patients diagnosed with aggressive lymphoma and their matched comparators in Sweden and Norway, stratified by sex and treatment group with p-values for differences in CIFs.
